# Miniaturized 0.13-μm CMOS Front-End Analog for AlN PMUT Arrays

**DOI:** 10.3390/s20041205

**Published:** 2020-02-22

**Authors:** Iván Zamora, Eyglis Ledesma, Arantxa Uranga, Núria Barniol

**Affiliations:** Departament d’Enginyeria Electrònica, Universitat Autónoma de Barcelona, 08193 Bellaterra, Spain; ivan.zamora@uab.es (I.Z.); eyglis.ledesma@uab.es (E.L.); arantxa.uranga@uab.es (A.U.)

**Keywords:** ultrasound, PMUT, high-voltage (HV) transmitter, low-voltage receiver (RX) amplifier, ultrasound application-specific integrated circuit (ASIC), monolithical integration, CMOS, MEMS

## Abstract

This paper presents an analog front-end transceiver for an ultrasound imaging system based on a high-voltage (HV) transmitter, a low-noise front-end amplifier (RX), and a complementary-metal-oxide-semiconductor, aluminum nitride, piezoelectric micromachined ultrasonic transducer (CMOS-AlN-PMUT). The system was designed using the 0.13-μm Silterra CMOS process and the MEMS-on-CMOS platform, which allowed for the implementation of an AlN PMUT on top of the CMOS-integrated circuit. The HV transmitter drives a column of six 80-μm-square PMUTs excited with 32 V in order to generate enough acoustic pressure at a 2.1-mm axial distance. On the reception side, another six 80-μm-square PMUT columns convert the received echo into an electric charge that is amplified by the receiver front-end amplifier. A comparative analysis between a voltage front-end amplifier (VA) based on capacitive integration and a charge-sensitive front-end amplifier (CSA) is presented. Electrical and acoustic experiments successfully demonstrated the functionality of the designed low-power analog front-end circuitry, which outperformed a state-of-the art front-end application-specific integrated circuit (ASIC) in terms of power consumption, noise performance, and area.

## 1. Introduction

Ultrasound, since it was discovered, has been a widely used tool for multiple applications such as medical echography and nondestructive testing. However, today the ultrasound sensing market is showing an impressive resurgence: new applications along with improved manufacturing capabilities and advanced technological readiness are driving the growth of micromachined ultrasound transducers (MUTs). Volumetric medical imaging [1], in vivo and in vitro neuromodulation ultrasound [2], fingerprint sensing [3], and gesture recognition [4] are some new applications based on MUTs.

Nowadays, two different micromachined ultrasound transducers can be found in the literature [5]: the first one is based on a capacitive resonant element (CMUT) that consists of a thin metallized suspended membrane over a cavity with a rigid metallized substrate. A DC voltage is applied between two electrodes, which causes the membrane to deflect toward the substrate due to the electrostatic force. Therefore, an ultrasound can be generated in the surrounding medium from the vibration of the membrane when AC voltage is imposed. CMUTs, although they can be integrated over a preprocessed complementary-metal-oxide-semiconductor (CMOS) substrate, have the following drawbacks: they require a large DC bias, different transmission and reception arrays may be required for imaging applications, and they have higher equivalent capacitance. The second transducer is based on piezoelectric materials (PMUTs), in which (in contrast to CMUTs) the deflection of the membrane is produced by the lateral strain generated from a piezoelectric actuation, whereby the membrane must include at least one piezoelectric layer as well as a passive elastic layer. Although PMUTs have low electromechanical coupling coefficients and produce little output acoustic pressure with narrow bandwidths compared to CMUTs, PMUTs do not require DC bias, they have fewer geometric and design constraints, and the same transmission and reception arrays with equal coupling may be used for imaging applications [5]. Additionally, the capability of using a low thermal process for the piezoelectrical material, such as aluminum nitride (AlN), allows for the possibility of monolithically integrating PMUTs on CMOS.

**Figure 1:**
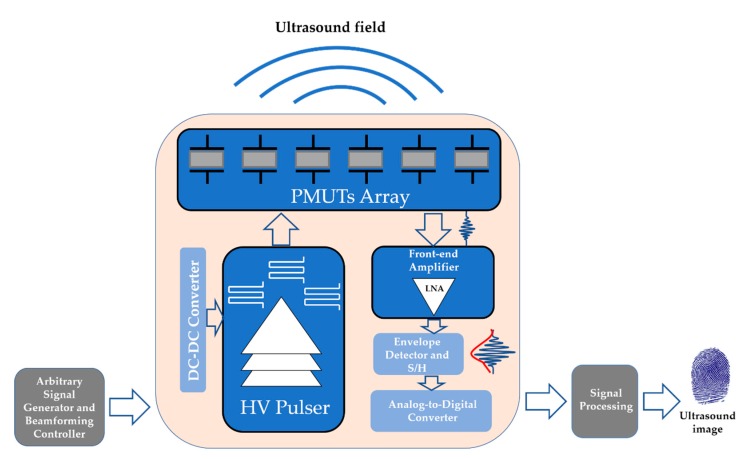
Image of an ultrasonic system block diagram.

Figure 1 shows a typical block diagram for ultrasound imaging applications using PMUTs. On the transmission side, the high-voltage (HV) pulser is able to drive the ultrasound transducer to emit enough acoustic pressure. In order to focus on a specific point, a transmission beamforming controller is used to generate the respective delays. A high-voltage DC–DC Converter may be required to generate the supply voltage of the HV pulser. On the reception path, isolation switches included in the front-end amplifier functional block are used to isolate the HV present in the transmission of low-voltage circuits used in the reception. Due to the weak electrical signals generated by the PMUTs, a low-noise amplifier (LNA) is employed to amplify these signals. After amplification, the amplitudes of these signals are extracted by an envelope detector and captured by a sample-and-hold circuit, which will later be digitized and processed. In order to obtain an image, most ultrasound measurements are based on the pulse echo method, where a generated short ultrasound pulse is propagated in a specified direction and is partly reflected wherever there is a change in the acoustic properties of the medium. The reflected ultrasound echo is processed in order to obtain information about distance, shape, and the physical properties of the target. An image is formed by mapping the echo strength versus travel time (proportional to the distance).

The integration of PMUTs and CMOS to form a single chip allows for a reduction in parasitic elements, size, weight, and power consumption of the overall system. Recent works [3,6,7,8] have presented a single-chip ultrasonic fingerprint sensor based on PMUTs that are directly bonded to a CMOS readout application-specific integrated circuit (ASIC) using Al-Ge eutectic bonding. This integration strategy (presented by Reference [8]) is a considerable improvement over the wire bonding method and reduces the electrical parasitic elements, but it is a very expensive process, and due to the presence of special eutectic bonding, the fill factor is affected. In order to overcome these drawbacks, the Silterra foundry has implemented a MEMS-on-CMOS platform, where an AlN-based PMUT can be fabricated over CMOS preprocessed wafers. This monolithic integration of a PMUT over the CMOS reduces fabrication complexity, minimizes the interconnections between PMUT neighbors and the CMOS electronic circuitry, and reduces the parasitic elements; consequently, a higher fill factor and improved signal integrity are possible. In this work, we present the design of a 0.13-μm CMOS front-end transmitter and receiver using the MEMS-on-CMOS technological platform from Silterra [9]. This paper is organized as follows: Section 2 describes the design of the system, and in particular, Section 2.1 presents the ultrasonic transducer; Section 2.2 describes the high-voltage transmitter circuitry and its simulation results; Section 2.3 describes two of the front-end amplifiers that are most used to convert the electric charge generated by PMUT into voltage; and Section 2.4 compares both reception (RX) approaches, making use of the simulation results. Section 3 presents the electrical characterization of the circuitry, and Section 4 shows the acoustic experiments, including an ultrasound image.

## 2. Front-End Analog Circuit Design

### 2.1. Ultrasonic Transducer

As proof of concept for the integrated front-end circuitry, an 80-μm/side AlN PMUT in a 6 × 6 array configuration was used [10]. The electrical capacitance of a single PMUT was estimated at around 200 fF, adding up to a total of 1.2 pF per row. The obtained resonance frequency in fluorinert (FC-70) was in the 3-MHz range, with a bandwidth around 1 MHz [10].

### 2.2. High-Voltage Transmitter

The transmitter circuit is in charge of the generation of a large voltage signal in order to excite the transducer at its resonance frequency (*f_r_*) and provide enough acoustic pressure for a high signal-to-noise ratio in the echo measurements. We set the needed actuation voltage to a maximum voltage of 32 V to guarantee enough output pressure [10]. Additionally, and taking into account the fact that the square wave increases the effective amplitude 1.27 times compared to a sine wave, we chose a squared-wave transmitter circuit with a 32-V peak voltage and with rising and falling times less than 100 ns to guarantee at least a 50% duty cycle and a 3-MHz driving frequency (according to the expected PMUT resonance frequency in fluorinert).

Previous transmitter circuits [11,12] have used a conventional level shifter that consists of an nMOS differential pair and cross-coupled pMOS transistors. This structure, although it is very simple, has two main problems. On the one hand, the input transistors (nMOS) need to be large in order to overpower the pMOS load when the inputs change, resulting in high input capacitance and therefore limiting the maximum operation speed. On the other hand, a large crowbar current flows when both the nMOS and pMOS transistors are conducting, affecting the efficiency and power consumption of the system. In Reference [13], a charge-recycling HV pulser (CRHV) was presented in order to improve the power consumption, although it needs four control signals with different logic levels per channel, which makes the circuitry more complex.

**Figure 2:**
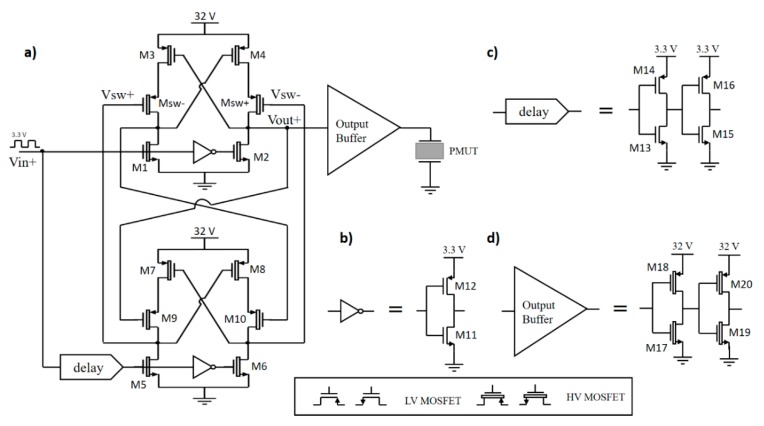
Schematic of the high-voltage transmitter. (**a**) Complete architecture to excite the piezoelectric micromachined ultrasonic transducer (PMUT). (**b**) An inverter circuit. (**c**) Cascaded inverter used as a delay element and (**d**) an output buffer using an inverter string to drive large capacitive loads.

In order to solve the issues mentioned above, we chose the circuit proposed by Reference [14]. This circuit has the basic structure of a conventional level shifter (see Figure 2), but introduces additional high-voltage devices (Msw+ and Msw-) in a series with latching transistors (M3 and M4) in order to avoid the crowbar current. These new transistors work as switches controlled by the high-voltage signals Vsw+ and Vsw-, which inhibit the current of M3 and M4 when the nMOS devices (M1 and M2) are driving the current. The control signals (Vsw+ and Vsw-) are obtained from the output of a second dynamic level shifter, where its input signal is delayed with respect to Vin+ [15].

**Figure 3:**
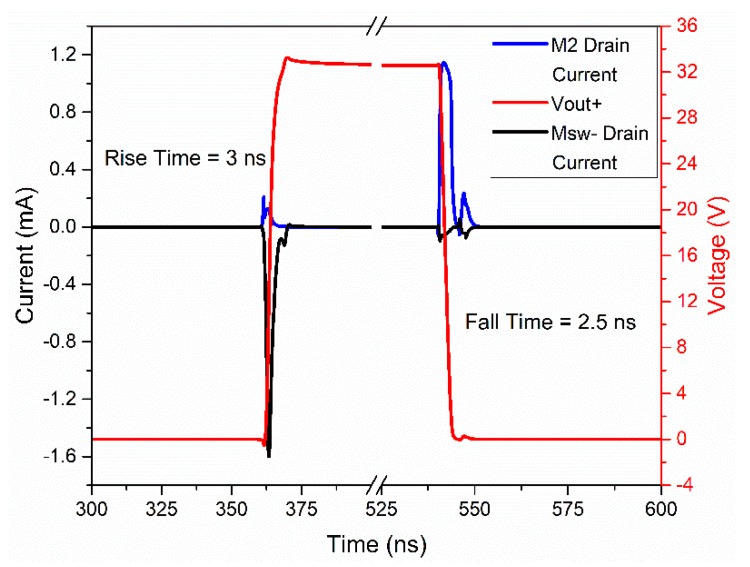
Voltage and source and sink currents in the output node (Vout+) [15].

In order to optimize the maximum operation speed and dynamic power consumption, a proper dimensioning of the nMOS and pMOS transistor sizes was done. Figure 3 shows the simulated source and sink currents and the rise and fall times in the output node (Vout+ node). It can be seen how the presence of the Msw+ and Msw- devices allows for the removal of the crowbar current. A high-voltage inverter buffer was designed to drive large capacitive loads. The dimensions of the MOSFETs selected for this circuit are shown in Table 1.

**Figure 4:**
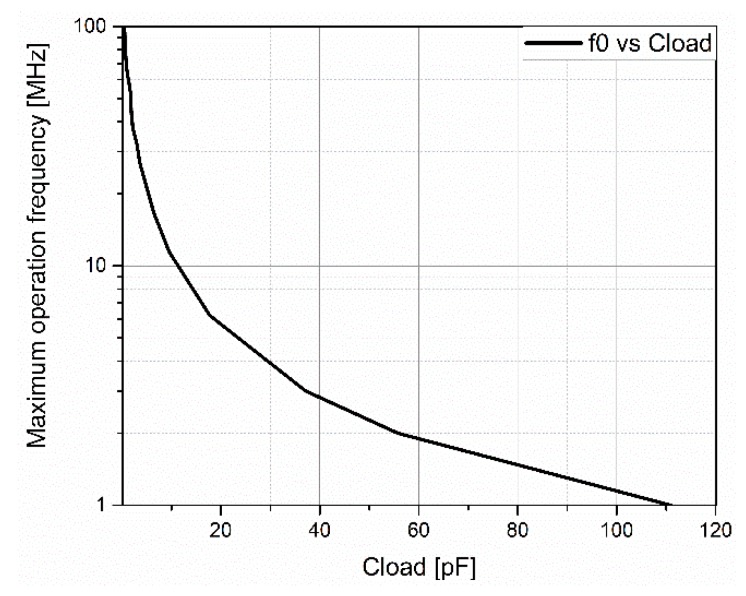
Maximum transmitter operating frequency as a function of load capacitance (simulation results).

In most imaging ultrasound applications, the transducer is an array of PMUTs. To obtain enough acoustic pressure level, several PMUTs of the array emit simultaneously, forming a transmit channel. In order to reduce the size of the die, one HV transmitter is used per channel, and therefore it has to be able to drive a large load capacitance at the resonance frequency. Figure 4 shows the simulation results of the maximum operation frequency of our HV transmitter, taking into account the designed output buffer, as a function of the load capacitance. Taking into account that the capacitance associated with an individual PMUT was in the range of 200 fF, we could make Figure 4, which indicates the maximum number of PMUTs simultaneously actuated at each frequency (e.g., in the case of 3 MHz, the maximum capacitance corresponded to 37 pF, which is equivalent to 185 individual PMUTs, a reasonable number for a linear array configuration).

With this circuit, 2.9 ns of a 10–90% rise/fall time was obtained for 1.2 pF (equivalent to one row of a 6 × 6 PMUT array) of load capacitance, achieving the design requirements.

### 2.3. Front-End Amplifier

The operation of PMUTs as a sensor is governed by the direct piezoelectric effect, where an electrical charge is generated when the acoustic wave arrives at the PMUT. In order to measure this electric charge, it was necessary to design a low-noise amplifier (LNA) at the first stage of the front-end PMUT receiver circuit that was a tradeoff between power supply, noise performance, gain, bandwidth, dynamic range, and die size. Transimpedance amplifier topologies (TIAs) are widely used to amplify the weak signal generated by relatively high-impedance CMUTs [12,16]. Nevertheless, piezoelectric transducers (PZTs and PMUTs) with similar sizes present much lower equivalent impedance around their resonance frequency, whereas this topology suffers from a low noise/power tradeoff when it is chosen as a front-end amplifier for PMUT devices. A capacitive feedback voltage amplifier (CFVA) with a split-capacitor feedback network has been a very common topology in readout integrated circuits (ICs) for PZT transducers [17,18] (in order to sense the transducer’s voltage rather than its electric charge). Nevertheless, this topology suffers from a large area due to the use of several capacitors. This work presents a comparative analysis of two of the most used front-end amplifier configurations in sensors based on PMUTs: a voltage amplifier (VA) based on capacitive integration [11,13,15] and a charge-sensitive amplifier (CSA) [3,19].

#### 2.3.1. Voltage Amplifier (VA) Description

**Figure 5:**
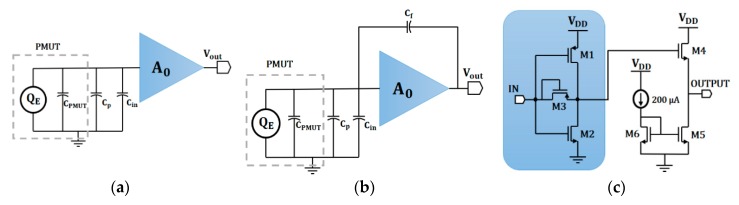
PMUT front-end amplifier configurations: (**a**) a voltage amplifier (VA) based on capacitive integration, (**b**) a CSA amplifier, and (**c**) a proposed schematic for the open-loop voltage amplifier, A_0_, for the VA and CSA receivers.

Figure 5a shows a scheme of a front-end amplifier based on capacitive integration. The accumulated electric charge on the input equivalent capacitance (the parallel between C_PMUT_, the electric capacitance of the PMUT; C_in_, the input capacitance of the voltage amplifier; and C_p_, all of the parasitic capacitances) is converted into voltage and is later amplified by the designed voltage amplifier. This relationship can be expressed by Equation (1), where A_0_ is the open-loop amplifier voltage gain and Q_E_ is the electric charge generated by the PMUT:

(1) $$V_{\mathrm{out}}=A_{0}\frac{Q_{E}}{C_{\mathrm{PMUT}}+C_{P}+C_{\mathrm{in}}}\;$$

To find a tradeoff between power consumption, gain, bandwidth, and area, a single-ended input self-biased push–pull configuration was selected as the VA topology (see Figure 5c). With this configuration, a smaller possible area could be used for the amplifier, which allows for a pitch-matched circuit with a single PMUT.

The input-referred noise current of this topology was derived from a small-signal equivalent model considering all parasitic capacitance (see Equation (2)):

(2) $$i_{n,\mathrm{in}}\left(s\right)=\frac{{\mathrm{sC}}_{\mathrm{in}}\left(C_{f}+C_{\mathrm{in}}\right)\left(i_{n,n}+i_{n,p}\right)\left[1+\frac{s\left(C_{f}+C_{\mathrm{out}}\right)}{g_{\mathrm{ds},n}+g_{\mathrm{ds},p}}\right]}{A_{0}\left[C_{f}\left(g_{m,n}+g_{m,p}\right)+C_{\mathrm{in}}\left(g_{\mathrm{ds},n}+g_{\mathrm{ds},p}\right)\right]\left(1-\frac{{\mathrm{sC}}_{f}}{g_{m,n}+g_{m,p}}\right)\left[1+\frac{s\left(C_{\mathrm{in}}C_{f}+C_{\mathrm{out}}C_{f}+C_{\mathrm{out}}C_{\mathrm{in}}\right)}{C_{f}\left(g_{m,n}+g_{m,p}\right)+C_{\mathrm{in}}\left(g_{\mathrm{ds},n}+g_{\mathrm{ds},p}\right)}\right]}$$

where i_n,n_ and i_n,p_ are the square root of the mean square current noise for nMOS and pMOS transistors; g_m,n_ and g_m,p_ are the transconductance for the M1 and M2 transistors, respectively; g_ds,n_ and g_ds,p_ are the channel conductance of M1 and M2, respectively; C_f_ is the feedback capacitance (in this topology, the sum of gate-to-drain capacitances); and C_out_ is the output capacitance of the circuit (approximately the load capacitance). Due to the small current generated by the PMUT (nanoamperes), the minimization of amplifier noise is a must. In order to minimize the input current noise and maximize the transimpedance gain, the pMOS M1 and nMOS M2 transistors were correctly sized. To maximize the dynamic range, the operation point of the amplifier was fixed to V_DD_/2 by a pMOS M3 transistor connected between the input and output (operating in the subthreshold region, which functions as very high-impedance resistance) [20]. A source follower buffer was added to match the output impedance to 50 Ω for testing.

#### 2.3.2. Charge-Sensitive Amplifier (CSA) Description

The second approach is the charge-sensitive amplifier shown in Figure 5b, where the output voltage, given by Equation (3), only depends on the feedback capacitance (C_f_), considering that the input impedance of the amplifier is much smaller than the equivalent impedance given by PMUT and the parasitic elements:

(3) $$V_{\mathrm{out}}=\frac{Q_{E}}{C_{f}}.$$

Since the input impedance is proportional to 1/(C_in_ + C_f_*A_0_), it is necessary to choose an amplifier topology that guarantees a high open loop gain A_0_ (with minimum dimensions) to decrease Cin and the area. This reduction in the input capacitance impedance allows for the use of a low-feedback capacitance, C_f_, thus achieving a maximum charge transfer and SNR. Therefore, the same single-ended input self-biased push–pull configuration used in the VA was the right selection.

In order to select the C_f_, we took into account the input referred noise, the charge transfer from the PMUT to the feedback capacitance, and the gain. The input-referred noise current of this configuration is given by the following expression:

(4) $$i_{n,\mathrm{in}}\left(s\right)=\frac{s\left(C_{f}+C_{\mathrm{in}}\right)\left(i_{n,n}+i_{n,p}\right)}{\left(g_{m,n}+g_{m,p}\right)\left(1-\frac{{\mathrm{sC}}_{f}}{g_{m,n}+g_{m,p}}\right)}$$

**Figure 6:**
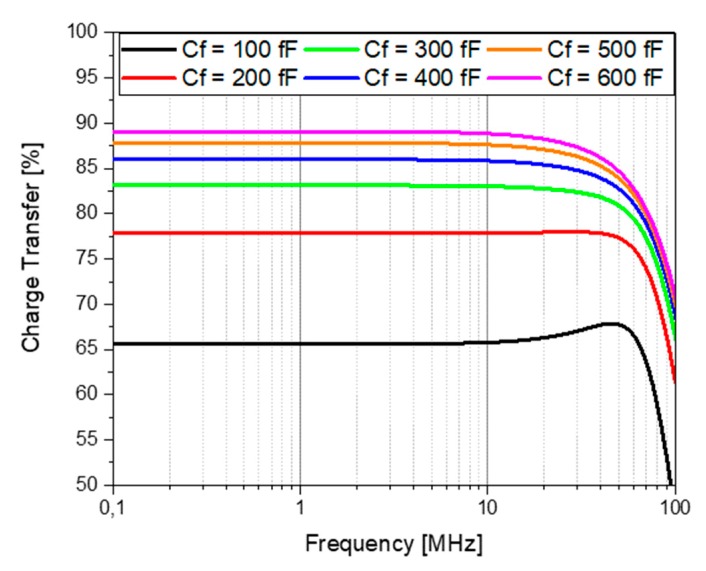
Charge transfer obtained for different feedback capacitances (C_f_).

This equation shows that the input noise current is directly proportional to C_f_, and therefore the feedback capacitance must be selected to be as low as possible. In order to perform good signal processing, at least 85% of the charge transfer must be guaranteed. Considering a C_PMUT_ = 1.2 pF (equivalent to one row of six 80-μm PMUTs [10]), a parametric AC analysis was performed to compute the charge transfer. Figure 6 shows that for a C_f_ higher than 400 fF, the amplifier behaves as a CSA and guarantees at least an 85% charge transfer. Therefore, in order to maximize the output voltage given by Equation (3) and to find a tradeoff between noise performance and charge transfer, 400 fF was selected as the feedback capacitance. In this case, a pMOS M3 transistor was used to fix the operation point and was dimensioned (without affecting the bias) to avoid any leakage current that could charge C_f_, leading to saturation of the amplifier. The same source-follower buffer was used to match the output impedance to 50 Ω.

**Figure 7:**
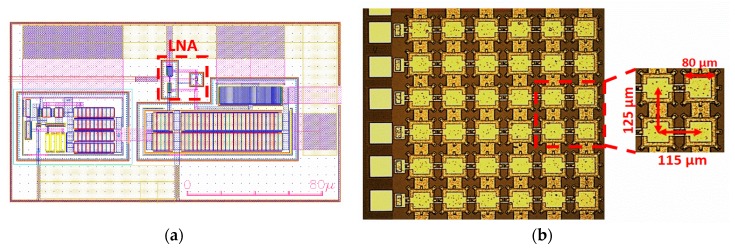
(**a**) Layout of the LNA (VA topology) with an output buffer. The LNA dimensions are 23 μm of width and 25 μm of length. (**b**) Optical image of a 6 × 6 (square) 80-μm/side PMUT array.

In Figure 7, the final layout of the LNA based on a VA is shown together with an optical image of the PMUT array, demonstrating its capabilities for a minute area and a pitch-matched system (for the CMOS receiver).

### 2.4. Front-End Amplifier Simulation Results

All simulations were done with 14 pF and 50 Ω as the load impedance (input impedance of the oscilloscope). The PMUT was modeled as a current source in parallel with its electrical capacitance. The assumed operation frequency was the resonance frequency of the PMUT array in liquid (3 MHz), which was given by Reference [10].

#### 2.4.1. Transimpedance Gain and Input Impedance

**Figure 8:**
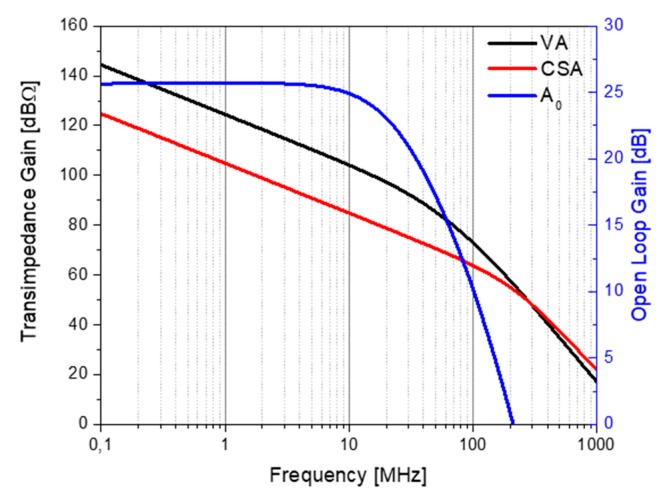
Frequency response of both amplifiers (VA and CSA) (left axis) along with the push–pull open-loop response of A_0_ (right axis).

Figure 8 shows the transimpedance gain of both approaches (the voltage amplifier and charge-sensitive amplifier) and the voltage open-loop gain A_0_. It can be seen that the VA amplifier had a higher transimpedance gain (≈23 dB Ω), since the output voltage of the VA depended not only on the amplifier open-loop gain A_0_, but also on the integration of the current in the input capacitance. The CSA transimpedance gain depended only on the value of the feedback capacitance (it needs to be lower than the total VA input capacitance). However, the VA amplifier frequency response was limited by the open loop bandwidth (21.7 MHz). In the case of the CSA, its frequency behavior was limited by its input impedance and the equivalent impedance of PMUTs, plus the parasitic capacitance. At the frequency where both impedances were similar (around 140 MHz), the electric charge generated by the PMUT was divided, and therefore the CSA stopped behaving like a charge-sensitive amplifier.

**Figure 9:**
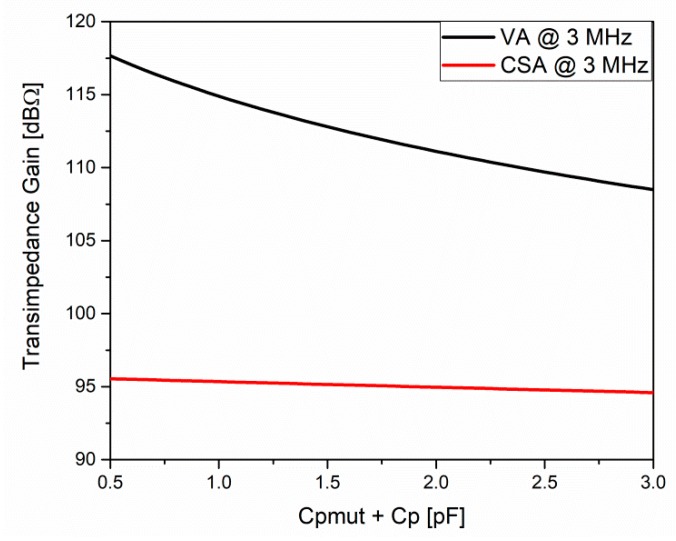
Dependence of the transimpedance gain at 3 MHz, with equivalent capacitance given by the PMUT plus parasitic capacitances.

Figure 9 shows the dependency of the transimpedance gain in both cases (VA and CSA amplifiers), with the PMUTs and parasitic capacitances. As expected given Equations (1) and (3), the VA had a steeper slope (ΔTIGain = 7.5 dBΩ), since it amplified the generated input voltage through the integration of the PMUT current in all of the capacitances present in the input of the amplifier. Instead, the transimpedance gain of the CSA had a linear dependency with the lower slope (ΔTIGain = 0.8 dBΩ), but still the VA had more gain.

#### 2.4.2. Noise Performance and Dynamic Range

**Figure 10:**
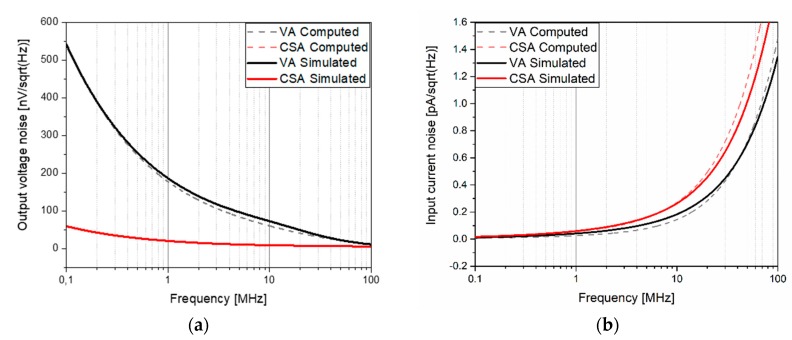
Computations using Equations (2) and (4) and the simulated RX amplifiers’ noise spectral density. (**a**) Output voltage noise and (**b**) input current noise.

To compare the noise performance of both amplifiers, the output noise voltage (V_n,out_) and the input noise current (I_n,in_), defined by Equations (2) and (4), were taken into account. The presence of the feedback capacitor in the CSA amplifier caused its output impedance to be smaller than the VA output impedance, causing the output voltage noise to be smaller (see Figure 10). However, due to the big difference between their transimpedance gains, the input noise current was lower for the VA than for the CSA (0.08 pA/√Hz and 0.12 pA/√Hz, respectively, at a 3-MHz frequency).

**Figure 11:**
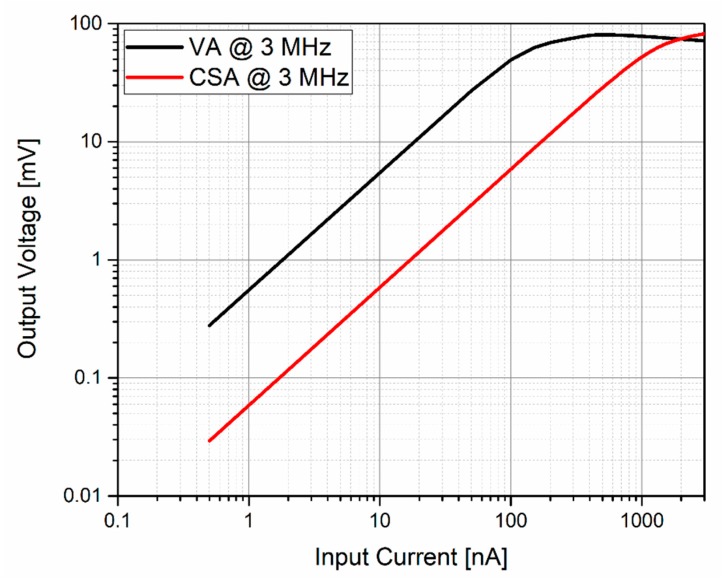
Simulated dynamic range at 3 MHz.

Figure 11 shows the simulated dynamic range for a 3-MHz input signal. A maximum input current of 100 nA and 1 μA for the VA and CSA were obtained, respectively. This great difference was expected due to the big difference between their gains. In these results, the CSA provided a dynamic range 10 times higher than the VA amplifier, allowing it to amplify PMUT signals 10 times larger than those amplified by the VA.

## 3. Electrical Characterization

### 3.1. High-Voltage Transmitter

**Figure 12:**
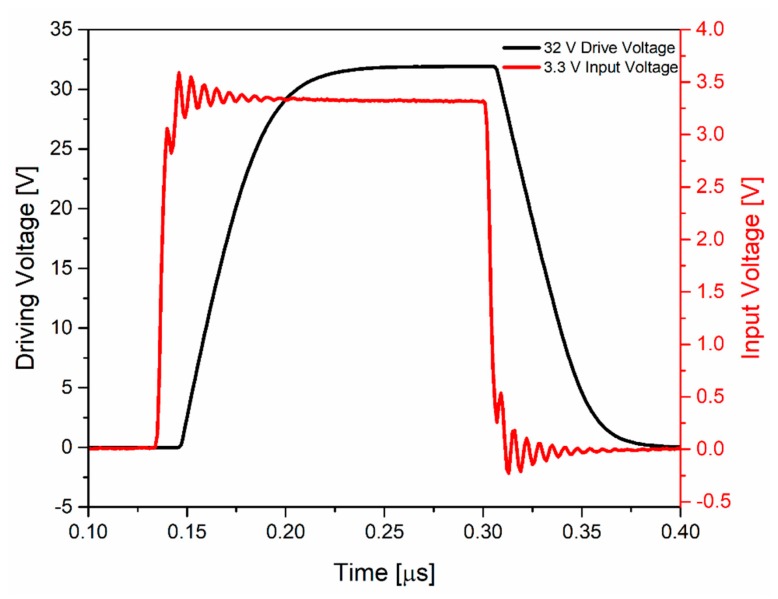
Waveform of the HV transmitter input and output.

The HV transmitter circuit was tested using a signal generator and an oscilloscope. The circuitry, operated from two power supplies (3.3 V and 32 V) and with a 3.3-V-squared input signal, generated output pulses up to 32 V. Figure 12 shows the input and output waveforms of this circuit, which achieved a rise/fall time of 47.5 ns. Using 14 pF as the load capacitance from the oscilloscope plus 3 pF due to the bonding pad, printed circuit board (PCB) pad, and connectors, this value differed by 6.36 ns compared to the simulation result. This difference could have been due to the estimation of the load capacitance (parasitic capacitance) as well as due to process variations. For this estimated load capacitance, the HV transmitter operated at a maximum frequency of 5 MHz, achieving a 10–90% rise time and a 50–50% latency of 47.5 ns and 31 ns, respectively.

The static power consumption was negligible, and the dynamic power consumption, defined as C_L_V^2^f, was 3.68 mW for a 1.2-pF load capacitance.

### 3.2. Front-End Amplifiers

The RX amplifiers were characterized in terms of voltage–voltage gain for the VA, transimpedance gain for the CSA, and input current noise and output dynamic range for both of them.

**Figure 13:**
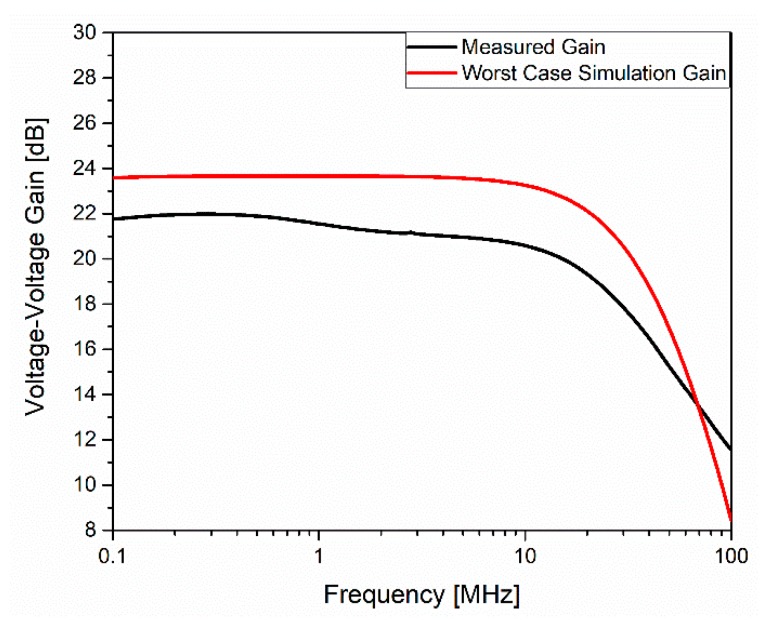
Transfer function of the VA amplifier.

Figure 13 shows the measured and postlayout simulation voltage–voltage gain of the voltage amplifier (VA). A measured bandwidth of about 22 MHz was achieved. Compared to the postlayout simulation and considering the technology variations, the simulated values were close to the experimental ones.

**Figure 14:**
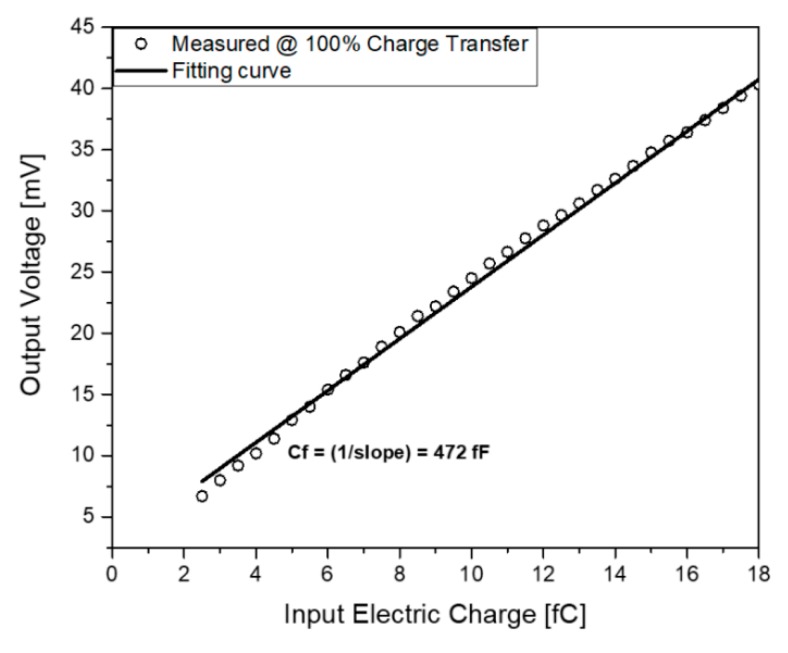
Tested results of the output voltage of the CSA.

In order to compute the gain of the CSA, we employed the charge injection method. A 100-fF capacitor connected in series at the CSA input was used as a test injection capacitor (C_TEST_). Small voltage steps (ΔV) applied to the C_TEST_ produced charge pulses at the CSA input with a value of C_TEST_ *ΔV. Figure 14 shows the output voltage measured at the buffer output for an input charge sweep from 2.5 fC to 18 fC (corresponding to voltage steps from 25 mV to 180 mV). According to Equation (3), the slope of this graph must be inversely proportional to the feedback capacitance, giving 472 fF with a 100% charge transfer. Taking into account this and using the designed feedback capacitance (Cf = 410 fF in the layout), the obtained charge transfer was 87%, close to the simulated results.

**Figure 15:**
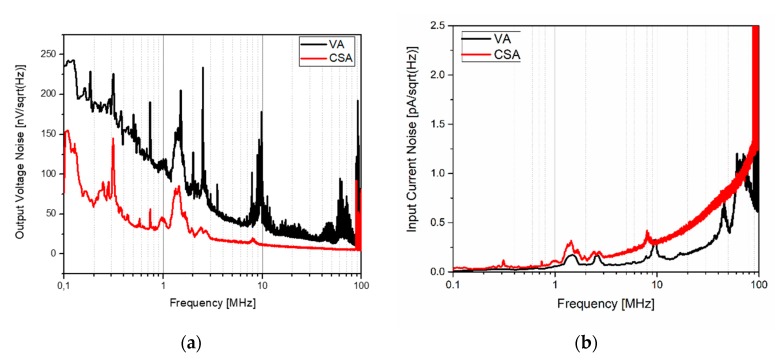
Measured spectral density noise: (**a**) output-referred voltage noise and (**b**) input-referred current noise.

The noise performance of both the VA and CSA amplifiers was measured by opening the input and measuring the output noise. The input noise was computed by dividing the measured output noise by the corresponding transfer function. The resulting spectral density noise is shown in Figure 15, which was in good agreement with the simulations. The measured output-referred voltage noise density was 60.68 nV/√Hz and 19.01 nV/√Hz at 3 MHz for the VA and CSA amplifiers, respectively. At the same frequency, the measured input-referred current noise was 68 fA/√Hz and 147 fA/√Hz for the VA and CSA, respectively. Taking into account the PMUT bandwidth (1 MHz in liquid), an input-referred integrated noise of 59.18 pA_rms_ and 142.7 pA_rms_ was obtained for the VA and CSA, respectively.

Considering that the reception sensitivity of the PMUT array [10] was 5.9 V/MPa, the measured noise equivalent pressure was 0.84 mPa/√Hz and 1.32 mPa/√Hz at 3 MHz. These values are very low for imaging applications, where the received echoes are in the order of kPa, so a good signal-to-noise ratio can be guaranteed.

**Figure 16:**
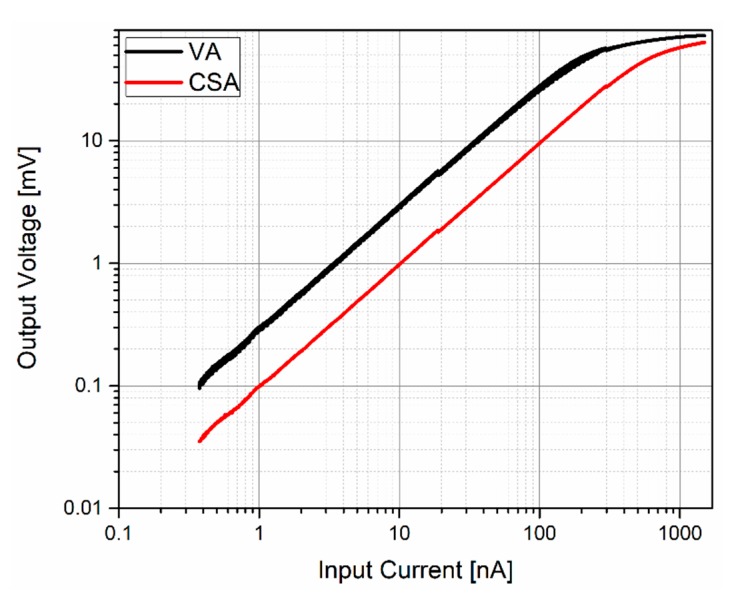
Dynamic range measurements: the VA amplifier (black) and the CSA amplifier (red).

Figure 16 shows the measurements of the VA and CSA output voltage for different input current levels at 3 MHz. The measured transimpedance gain was 109.22 dBΩ and 99.57 dBΩ, respectively. An input-measured dynamic range of 69 dB and 71 dB was achieved, where the previous measured integrated input noise was taken as the minimum detectable current.

## 4. Acoustic Characterization

**Figure 17:**
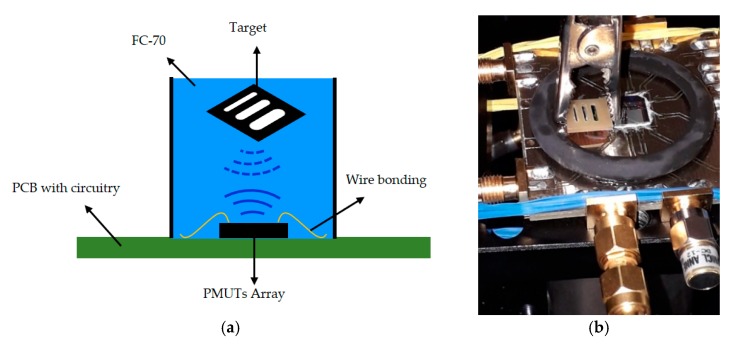
Set-up for acoustic characterization using an FC-70: (**a**) the schematic set-up; (**b**) an optical image of the experimental set-up using a grating phantom.

**Figure 18:**
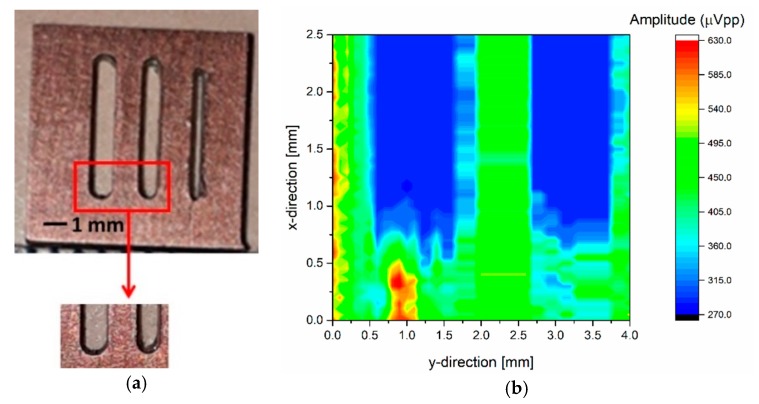
(**a**) Grating phantom and (**b**) an ultrasonic image of a phantom section.

To validate the analog front-end CMOS in its intended application, an acoustic characterization was done using fluorinert (FC-70, where the mass density is ρ = 1940 kg m^−3^ and the sound velocity is c = 690 m/s) as a propagation medium. The set-up is shown in Figure 17, where a grating phantom was introduced in a small pool, confining the FC-70 to approximately 2.1 mm over the 6 × 6 PMUT array surface. One row of this array was excited by the designed HV transmitter (four cycles at 3 MHz with a 32-V peak-to-peak), and another row was dedicated to reception using the proposed VA as an LNA front-end amplifier. In order to obtain a 2D image, the grating phantom was shifted in the × direction and *y* direction, with steps of 50 μm and 100 μm, respectively. Figure 18b shows an image of a section of this phantom (see Figure 18a). The blue bars correspond with the slots, with a width close to 1.1 mm and 1 mm, as was expected (physical width), demonstrating the capability of the system to make an ultrasonic image.

## 5. Discussion

In this work, we present the design and characterization of two functional blocks of an imaging ultrasound system: an HV transmitter and a front-end receiver amplifier. In addition, we compared two of the most used front-end amplifiers in sensor-based PMUTs.

In this section, we will compare these proposals to prior work, taking into account all of the obtained results.

### 5.1. HV Transmitter

Table 2 summarizes and compares our transmitter to prior reported works. The performance of the HV transmitter was measured in terms of its speed, area, power consumption, and load capacitance. In order to compare different systems, we defined a figure-of-merit (FOM) that takes into account all of the key parameters that intervene in the transmitter’s performance (see Equation (5)):

(5) $${\mathrm{FOM}}_{\mathrm{TX}}\left[\frac{\mathrm{mA}}{{\mathrm{mm}}^{2}}\right]=\frac{V_{\mathrm{MAX}}\left[V\right]{*C}_{L}\left[\mathrm{pF}\right]}{t_{\mathrm{rise}}\left[\mathrm{ns}\right]*\mathrm{area}\left[{\mathrm{mm}}^{2}\right]},$$

where C_L_ corresponds to the nominal load capacitance, V_MAX_ is the maximum pulsed output voltage, and t_rise_ is the rise time. Our designed HV transmitter achieved the best FOM_TX_ (1018 mA/mm^2^), which translated to the best electrical performance with the highest integration density. This performance was attributable in part to the small area that the transmitter has.

### 5.2. Front-End Receiver Amplifier

To compare both presented LNA topologies to the state-of-the-art topologies, we also defined a figure-of-merit that includes the main parameters of the LNA design. Taking into account the gain, input referred noise, power consumption, operation frequency, and area, we defined the following FOMs:

(6) $${\mathrm{FOM}}_{\mathrm{RX}\_1}\left[\frac{\mathrm{MHz}}{V^{2}{*A*\mu m}^{2}}\right]=\frac{\mathrm{Gain}\left[\frac{V}{V}\right]*\sqrt{\mathrm{frequency}\left[\mathrm{MHz}\right]}}{\mathrm{input}\;\mathrm{voltage}\;\mathrm{noise}\;\left[\frac{\mathrm{nV}}{\sqrt{\mathrm{HZ}}}\right]*\mathrm{Power}\;\mathrm{Consumption}\;\left[\mathrm{mW}\right]*\mathrm{area}\;\left[{\mathrm{mm}}^{2}\right]},$$

(7) $${\mathrm{FOM}}_{\mathrm{RX}\_2}\left[\frac{\mathrm{Hz}}{{\mathrm{mA}}^{3}*{\mu m}^{2}}\right]=\frac{\mathrm{Transimpedance}\;\mathrm{gain}\left[\frac{V}{A}\right]*\sqrt{\mathrm{frequency}\left[\mathrm{MHz}\right]}}{\mathrm{input}\;\mathrm{current}\;\mathrm{noise}\;\left[\frac{\mathrm{pA}}{\sqrt{\mathrm{HZ}}}\right]*\mathrm{Power}\;\mathrm{Consumption}\;\left[\mathrm{mW}\right]*\mathrm{area}\;\left[{\mathrm{mm}}^{2}\right]},$$

where Equation (6) was used to compare the VA and CFVA topologies and Equation (7) was used to compare the CSA and TIA topologies. Table 3 summarizes the comparisons between the performance of the designed LNAs and that of the other amplifiers reported as being state-of-the-art, demonstrating competitive results. Taking the best computed FOM_RX_1,2_, the designed VA and CSA topologies achieved ~2.5× and ~45× higher improvement, respectively, guaranteeing the best electrical performance with a minimum area. These competitive FOM values were possible since our two LNAs (based on a self-biased push–pull amplifier) have a smaller area (almost by 10 times), which makes these designs good candidates to implement a pitch-matched system, where the LNA can be implemented just below its ultrasound transducer.

Finally, comparing the two presented LNA implementations in this work, the VA amplifier provided a remarkable improvement in terms of transimpedance gain and input-referred noise, achieving a higher signal-to-noise ratio. Nevertheless, the great dependency of the VA gain on the parasitic capacitance means that the CSA is the more useful option when the amplifier is not fully integrated with the PMUTs, since all of the parasitic capacitances are difficult to efficiently control, providing different gains for the same devices. The VA is more adequate when the amplifier can be directly connected to the sensor, as happens when there is a monolithic integration of the CMOS and PMUTs.

## 6. Conclusions

A CMOS circuit was designed and implemented using the novel CMOS-MEMS monolithic integration platform from Silterra in order to drive a 6 × 6 PMUT array. The system presented with competitive performance in comparison to state-of-the-art highlighting area and noise performance. A comprehensive comparison between two RX front-end amplifiers was done, showing that the VA amplifier based on capacitive integration provided remarkable improvements in general terms but was more sensitive to parasitic elements, which will be controlled with monolithic integration. This system was successfully applied in imaging experiments.

## Figures and Tables

**Table 1:** Dimensions of the MOSFET devices used in the transmitter.

MOSFETs	Aspect Ratio (W/L) (µm/µm)
M1, M2	20/5.75
M3, M4, Msw+, Msw-	10/4
M5, M6	10/5.75
M7, M8, M9, M10	4.7/4
M11	10/1
M12	25/1
M13	1/4
M14	2.5/4
M15	1/3
M16	2.5/3
M17	10/4.8
M18	25/4
M19	20/4.8
M20	50/4

**Table 2:** HV transmitter performance summary and comparisons.

Parameter	This Work	[13] (2019)	[21] (2017)	[3] (2016)	[16] (2016)
Process technology	0.13 μm HV CMOS	0.18 μm CMOS	0.18 μm HV BCD8-SOI	0.18 μm HV CMOS	TSMC 0.18 μm HV CMOS
Transducer	PMUT	PMUT	CMUT	PMUT	CMUT
Pulsed output voltage (V)	32	5/13.2	100	24	30
Nominal operation frequency (MHz)	3	5	10	14	5
Nominal load capacitance (pF)	1.2	N/A	9.2	2	2
Area (mm^2^)	0.013	N/A	0.09 ^1^	0.017 ^1^	0.016
Rise time (ns)	2.9	N/A	14	10.56 ^2^	6.6
FOM_TX_ (mA/mm^2^)	1018	-	730	267	568

^1^ These areas were estimated using a chip micrograph. ^2^ Obtained as t_rise_ = 2.2RC_L_, where R = Vmax/Imax.

**Table 3:** Measured LNA performance summary and comparisons.

Parameter	This Work		[13] (2019)	[18] (2018)	[16] (2016)	[11] (2015)	[17] (2015)
**Topology**	VA	CSA	VA	CFVA	TIA	VA	CFVA
Process technology	0.13 μm HV CMOS	0.13 μm HV CMOS	0.18 μm CMOS	0.18 μm HV-BCD	TSMC 0.18 μm HV CMOS	0.18 μm HV CMOS	0.18 μm CMOS
Transducer	PMUT	PMUT	PMUT	PZT	CMUT	PMUT	PZT
Power supply (V)	1.5	1.5	1.5	1.8	1.8	1.8	1.8
Power consumption (mW)	0.3	0.3	0.08	0.79	1.4	N/A	0.135
Area (10^−4^ mm^2^)	6	9	N/A	30 ^1^	280	310	60
Voltage–voltage gain (dB)	21.8	N/A	29/30/42/53 ^2^	18	N/A	N/A	−12/6/24
Transimpedance gain (dBΩ)	109.22 at 3 MHz	99.57 at 3 MHz	N/A	N/A	116/113.5 110/104	N/A	N/A
Bandwidth (MHz)	22	N/A	10	20	10.2/10.8 10.6/10.5	N/A	9.8
Input current noise (pA/√Hz)	0.08 at 3 MHz	0.15 at 3 MHz	N/A	N/A	0.41 @ 5 MHz	N/A	N/A
Input voltage noise (nV/√Hz)	7.1 at 3 MHz	N/A	N/A	7.9 at 5 MHz	N/A	11 at 0.22 MHz	5.9 at 4 MHz
Input dynamic range (dB)	69	71	90	75	N/A	N/A	81
FOM_RX_1_ (MHz/V^2^Aµm^2^)	16674	N/A	N/A	949	N/A	N/A	6633 ^3^
FOM_RX_2_ (Hz/mA^3^µm^2^)	N/A	4.1*10^9^	N/A	N/A	0.09*10^9 3^	N/A	N/A

^1^ This area was estimated from a chip micrograph. ^2^ Including a TGC amplifier as a second stage. ^3^ Computed considering its higher gain.
